# Current research status and progress in neuropsychological development of children with congenital heart disease: A review

**DOI:** 10.1097/MD.0000000000040489

**Published:** 2024-11-01

**Authors:** Shuantong Lin, Xiaojun Su, Dequan Cao

**Affiliations:** a Department of Anesthesiology, Pingshan District Central Hospital of Shenzhen, Guangdong Province, China.

**Keywords:** cognitive function, congenital heart disease, neuropsychological development

## Abstract

Children with congenital heart disease (CHD) are receiving widespread attention for their neuropsychological developmental issues, which include cognitive, adaptive, motor, speech, behavioral, and executive functioning deficits as well as autism spectrum disorders. Timely identification of risk factors influencing neuropsychological development and implementation of appropriate interventions are crucial for enhancing the neuropsychological outcomes of children with CHD, ultimately benefiting the children, their families, and society as a whole. This comprehensive review article aimed to explore the epidemiology, risk factors, assessment methods, and monitoring strategies of neuropsychological development in children with CHD. By providing a detailed examination of these factors, this review serves as a valuable resource for researchers and practitioners in the field, facilitating deeper understanding and more effective management of neuropsychological issues in this vulnerable population.

## 1. Introduction

Improvements in cardiac treatment techniques and perioperative management have further increased the survival rate of children with congenital heart disease (CHD), even severe CHD, which used to be challenging to save, is now treated with surgery at an early stage. Meanwhile, growing attention has been paid to postoperative neurodevelopmental issues in these children and it has become clear that neuropsychiatric developmental abnormalities are more frequent than expected. Understanding the etiology and influencing factors of neurodevelopmental abnormalities in children with CHD is vital for the timely identification and early implementation of appropriate interventions, which could be beneficial for timely individualized rehabilitation and promote long-term social development. This article elaborates on the epidemiology of CHD, factors influencing neurodevelopment, and assessment and monitoring methods in children with CHD.

## 2. Epidemiology

CHD is the most common congenital anomaly in newborns, with an incidence of approximately 0.6% to 1.3%,^[1]^ and is a leading cause of noncommunicable disease-related deaths in individuals under 20 years of age.^[2]^ With advancements in diagnostic and cardiac surgical techniques, as well as improvements in postoperative care, the global mortality rate of CHD has decreased from 7.1/100,000 in 1990 to 2.8/100,000 in 2019,^[3]^ with a perioperative survival rate for children with CHD exceeding 90%.^[4]^ While 95% of children with simple CHD and 75% to 90% of children with complex CHD can survive into adulthood,^[5]^ approximately 50% of children with CHD experience neurocognitive deficits and impairments.^[1,6]^

Nonindustrialized countries had unfavorable or worsening risks for recent periods and birth cohorts.^[2]^ In nonindustrialized countries, several specific factors may influence the prevalence of CHD, such as limited medical resources, malnutrition, environmental factors, and lack of healthcare and preventive measures. Nonindustrialized countries face challenges in the prevention and treatment of CHD.

## 3. Current status of brain development

Approximately 16% of children with CHD are born prematurely, with a 2-fold higher incidence of CHD among premature infants than among full-term infants.^[7]^ Children with CHD often exhibit abnormalities in brain structural development, including reduced brain volume, white matter softening, hippocampal developmental defects, and abnormal cerebral cortex development, resembling the characteristics found in premature infants.

Studies by Olshaker et al^[8]^ involving 46 children with CHD revealed that the cerebellar volume in these children was smaller than that in typically developing children, along with a decreased ratio of cerebellar to supratentorial brain volume. Paquette et al^[7]^ conducted diffusion tensor imaging scans of 21 premature infants with CHD (23–36 weeks gestational age), 28 full-term healthy infants (≥37 weeks), and 27 non-CHD premature infants (23–36 weeks). They discovered that premature infants with CHD exhibited increased corpus callosum attenuation signals, higher radial diffusivity, delayed myelination, and reduced neural signal transmission speed compared with full-term healthy infants and non-CHD premature infants.

Rollins et al^[9]^ performed brain magnetic resonance imaging (MRI) scans on 48 infants who had undergone surgery for biventricular CHD before 9 months of age and compared the results with those of 13 normal children of the same age group. They found that the overall brain volume of infants with CHD was reduced by 54 mL, white matter volume decreased by 40 mL, and brainstem volume decreased by 1.2 mL.

Reich et al^[10]^ conducted MRI scans of 33 children with complex CHD who underwent staged surgeries at 2 years of age and during school age (6–8 years), and compared the brain results with those of 8 normal children. Their findings indicated lower brain volume, gray matter volume, deep gray matter volume, and white matter volumes in children with CHD than in normal children.

Additionally, Latal et al^[11]^ evaluated 48 adolescents undergoing early cardiopulmonary bypass (CPB) surgery for CHD and found 10% lower total hippocampal volumes compared with controls. Reduced hippocampal volumes correlated with lower total intelligence quotient (IQ).

As the mortality rates of surgical procedures for CHD decrease, improvement in perioperative morbidity and quality of life have become key metrics of quality of care. Rehabilitation therapy (RT) could enhance and restore functional ability and quality of life in children with CHD. A retrospective study conducted by Ubeda Tikkanen et al^[12]^ involving 1379 patients < 18 years of age undergoing first palliative or full (complete) repair of a congenital heart defect revealed that 586 patients received at least 1 type of RT, neonates were most likely to receive RT (80%), non-neonates ≤ 2 years of age more often received RT than those older than 2 years.

Rehabilitation interventions are frequently received in the acute postoperative period following congenital cardiac operations. Though RT could be beneficial in improving functional outcomes in the long term, the proper use of RT still related to multiple factors: the underlying heart condition, cardiac surgery, complications, and medical treatment.

Children with congenital heart defects often exhibit significant cerebral anatomical abnormalities, which can impact the development of their nervous systems. Engaging in activities that promote neurological development can enhance brain function in these children, potentially improving long-term outcomes.

## 4. Neurodevelopmental manifestations

Neurodevelopmental disorders are the most common complications in children with CHD.^[13]^ During school age, they may exhibit cognitive, motor, linguistic, social, and psychological impairments^[14]^ that significantly affect academic performance, self-care abilities, quality of life, and social adaptation.^[13]^ A meta-analysis conducted by Feldmann M et al^[15]^ on 3645 children with CHD reported hypoplastic left heart syndrome and univentricular CHD cohorts performed significantly worse than atrial and ventricular septum defect cohorts in total IQ, which contained not only verbal IQ, but also performance IQ. An older age at assessment was associated with lower IQ scores in cohorts with transposition of the great arteries (TGA).

Children with congenital heart defects often experience significant delays in neurocognitive development compared to their typically developing peers. These delays are evident not only in intellectual development but also in specific executive functions. Targeted interventions tailored to the particular manifestations of CHD can be beneficial in improving academic performance during the school years.

## 5. Cognitive impairment

A retrospective study^[16]^ at Fuwai Hospital involving 89 children undergoing surgical treatment for CHD revealed that the overall cognitive function development of children with CHD generally lags behind that of healthy children. There was a significant correlation between younger age at the time of surgical treatment and delayed neurodevelopment. Brosig et al^[17]^ conducted Bayley Scale evaluations of 64 children undergoing surgical treatment for CHD at 2 and 4 years of age, showing that the rate of cognitive impairment in children with CHD tends to increase with age. Additionally, evaluation results at a younger age may underestimate the overall rate of cognitive impairment.

Surgery for CHDs is often necessary to improve cardiac function and overall health. However, the timing of surgery can have implications for neurological development. Therefore, the decision regarding the timing of surgery should be individualized for each child, taking into consideration factors such as the severity of the heart defect, the child’s overall health, and their neurocognitive development status. A multidisciplinary team, including pediatric cardiologists, cardiac surgeons, neurologists, and developmental specialists, should be involved in this decision-making process.

## 6. Motor dysfunction

A meta-analysis of 46 studies on motor function in children with CHD showed that the prevalence of mild to severe motor dysfunction in children with CHD ranged from 12.3% to 68.6%, with severe motor dysfunction ranging from 0% to 60%. Severe motor dysfunction is more common in infants and toddlers.^[18]^ A meta-analysis by Kaeslin et al^[19]^ on motor function in children with CHD undergoing open-heart surgery suggested that delayed motor development is the most prominent neurodevelopmental disorder in infants with CHD and that early motor intervention can improve motor development. A study by Farr et al^[20]^ on motor function in 1416 children with CHD indicated that the prevalence of gross motor dysfunction was 12% and fine motor dysfunction was 10%, requiring special education and interventions for children to adapt to the social environment. Sprong et al^[21]^ found in their evaluation using the Bayley Scale on 215 children with complex CHD that at 18 months of age, CHD children are more likely to have gross motor impairment than fine motor impairment (12% vs 0%), and motor impairments present at a younger age do not improve with age.

Abnormal brain anatomy in children with CHD can cause delayed motor function development. Although restorative training may improve motor function, its long-term effects still require further research validation. In addition to restorative training, comprehensive interventions might also be crucial for improving motor function development. These may involve nutritional support, psychological support, family education, and other measures to improve the children’s overall physical and mental health.

## 7. Language dysfunction

Fourdain et al^[22]^ found in their evaluation using the Bayley Scale, 49 children with CHD aged 12 to 24 months that speech development in children with CHD significantly lagged significantly behind that of normal children. Expressive language abilities were notably delayed, and poor gesture communication skills at 12 months predicted delayed expressive abilities at 24 months. In a longitudinal follow-up study over 5 years by Gaudet et al^[23]^ involving 55 children with complex CHD, it was observed that children with CHD could exhibit significant speech dysfunction as early as 2 years of age. Reduced speech function scores at 2 years of age could predict severe impairment in speech function at 5 years of age. A prospective follow-up study^[24]^ of 426 children who underwent corrective surgery for CHD in China, using the Griffiths Mental Developmental Scales to assess development 1 to 3 years post-surgery, showed that children with CHD had language item scores of 85.5 ± 21.7, which was significantly lower than that of normal children. The study also highlighted the urgent need to establish follow-up and neurodevelopmental assessment systems tailored for children with CHD.

Language development delays in children with CHD can have significant implications for their social interactions and overall quality of life. Effective communication skills are essential for forming relationships, participating in social activities, and accessing educational opportunities. Therefore, addressing language delays early on is crucial to mitigate potential social challenges and improve overall outcomes for these children.

## 8. Psychological disorders

Research by Nayar et al^[25]^ on the relationship between CHD and autism spectrum disorder (ASD) suggests that children with CHD have an increased likelihood of developing ASD, possibly indicating a genetic link between the 2 conditions. A case–control study by Sigmon et al^[26]^ involving 8760 children with CHD and 26,280 normal children revealed an increased likelihood of ASD in children with CHD (OR 1.32; 95% CI 1.10–1.59). Childhood ASD may lead to a higher prevalence of depression in later life. A cross-sectional study by Hong Kong scholars^[27]^ involving 96 adolescent CHD patients analyzed their psychological and quality of life outcomes, indicating a negative psychological impact of CHD on children, with an increased occurrence of depressive symptoms and reduced quality of life. Meta-analysis results by Leo et al^[28]^ on psychological interventions for depression in adolescent and adult CHD patients suggest that psychological interventions may reduce depressive symptoms compared to standard care. However, further research is needed to determine the optimal duration, management methods, and frequency of psychological interventions to maximize the benefits. Children with CHD present mainly with delayed motor and cognitive development before the age of 2 to 3 years.^[13]^ They exhibit lower IQ, executive function, and language abilities than their peers before school. During school age, they may show reduced fine motor and visual-motor functions, along with an increased incidence of psychological disorders and special education needs. Therefore, targeted functional training, specialized psychological interventions, and treatments play crucial roles in improving the long-term development of children with CHD.

## 9. Risk factors

### 9.1. Genetic factors

Chromosome abnormalities account for approximately 30% of CHD.^[25]^ 40% to 63.5% of children with Down syndrome (trisomy 21) have CHD, manifesting primarily as developmental delays, speech and language impairments, and motor impairments.^[29]^ Children with DiGeorge syndrome (22q11.2 deletion syndrome) exhibit reduced hippocampal volume, abnormal neuronal connectivity, spatial and emotional regulation impairments, and a tendency towards schizophrenia.^[30]^ Deletion of 16p11.2 spanning 593kb^[31]^ can increase the incidence of autism in children with CHD, whereas duplication of 17p13.3^[32]^ can lead to intellectual developmental abnormalities in children with CHD. Metabolic abnormalities, resulting from gene transcription and translation errors, can lead to neurodevelopmental disorders. Downregulation of FOXP2 can cause impaired speech function and decreased executive function, and FOXP2 is also implicated in the development of autism.^[33]^ Although apolipoprotein E is a major lipid transporter in the central nervous system that plays a crucial role in neural function recovery following traumatic brain injury, carrying the APOE-ε2 allele is significantly associated with increased behavioral issues, impaired social interactions, and restricted behavioral patterns.^[34]^

By exploring the shared genetic factors between CHD and ASD, researchers may uncover common pathways involved in both conditions. This could lead to a better understanding of the biological processes contributing to CHD development and progression.

### 9.2. Types of CHD

Different types of heart disease have varying effects on the neurodevelopment of affected children.^[5,6]^

Children with single ventricle (SV) physiology are at risk for neurodevelopmental impairment early in life and neurocognitive impairment as they age. Mechanisms that could contribute to increased potential for cognitive impairment may include baseline brain dysmaturation beginning in utero, subsequent acquired injury occurring in early childhood from staged surgeries and pathophysiologic factors related to SV physiology itself throughout the lifespan as new arrhythmias, heart failure, and other issues may develop.^[35]^

Hiraiwa et al^[36]^ conducted a prospective longitudinal study involving 27 children with SV or TGA and 48 healthy controls underwent three-dimensional MRI and neurodevelopmental assessment at around 3 years (preschool age) and at 9 years (school age), total brain volume and regional brain volumes remained significantly smaller in SV children than in TGA children at both time points, though the growth slope of total brain volume was not significantly different between the SV and TGA groups. A meta-analysis conducted by Bucholz et al^[37]^ revealed that Neurodevelopmental outcomes in children with hypoplastic left heart syndrome and single right ventricle anomalies remain poor, lower socioeconomic status and maternal education were associated with greater early delays in communication and problem-solving and progressive delays in problem-solving and fine motor skills over time.

Children with a total anomalous pulmonary venous connection^[38]^ and interrupted aortic arch,^[39]^ also exhibit significantly delayed neurodevelopment. Tetralogy of Fallot accounts for 7% to 10% of all CHDs,^[40]^ and children with tetralogy of Fallot often demonstrate impairments in executive function, cognition, adaptive function, language function, motor function, and 1 or more neurodevelopmental problems. Children with atrial septal defect^[41]^ and ventricular septal defect^[42]^ also frequently show varying degrees of neurodevelopmental delay.

Given the complexity and variability of CHD, including the diverse spectrum of anatomical and physiological abnormalities, further research is needed to better understand how these factors influence long-term neurodevelopmental outcomes. Longitudinal studies focusing on the neurodevelopmental trajectories of children with complex CHD can provide valuable insights into effective intervention strategies and optimize outcomes for these individuals.

## 10. Surgical factors

The relationship between the optimal age for surgical treatment in children with CHD and postoperative neurodevelopmental outcomes has not yet been conclusively established.^[43]^ Lim et al^[44]^ conducted a prospective cohort study of 45 children undergoing arterial switch operations for CHD, indicating that older age at surgery (>13 days) was identified as a risk factor for perioperative brain growth impairment and was associated with slower language development at 18 months. In a multicenter survey of 1416 children with CHD, O’Connor et al^[45]^ found that surgeries with a higher Aristotle Basic Complexity Score and Risk Adjustment in Congenital Heart Surgery-1 levels were more likely to result in delayed neurodevelopment and reduced quality of life postoperatively. Precise surgical techniques can expedite the recovery of children with CHD and reduce the risk of neurodevelopmental delays.^[46]^

Nathan et al^[47]^ evaluated 140 children with CHD using the Bayley Scale at 16 months of age, revealing a correlation between higher Technical Performance Scores and lower neurodevelopmental scores. Sengupta et al^[48]^ similarly suggested in their study of 784 children with CHD undergoing surgical treatment that higher Technical Performance Scores could predict both short- and long-term developmental impairments postoperatively.

In the treatment of CHD, the selection and monitoring of surgical classification and the technical skills of surgeons are crucial. Ensuring accurate surgical classification and selecting experienced and skilled surgeons for surgical operations can maximize the safety and recovery of children, and reduce adverse effects on their neurodevelopment. Additionally, systematic assessment and tracking of the neurodevelopment of children after surgery are necessary to timely identify and intervene in any potential problems and promote the comprehensive development of children.

## 11. Anesthesia factors

Although the impact of anesthetic agents on neurodevelopment has been validated in animal models, there is still no consensus on the effects of anesthesia in children with CHD.^[49]^ Follow-up studies by Andropoulos et al^[50]^ on 59 children with CHD undergoing surgery under general anesthesia indicated that postoperative cognitive dysfunction was related to prolonged exposure to high concentrations of inhaled anesthetics during surgery. In contrast, a retrospective study by Simpao et al^[51]^ on 100 children with CHD undergoing surgery under general anesthesia suggested that exposure to inhaled anesthetics was not associated with neurodevelopmental disorders in children with CHD postoperatively; however, higher doses of ketamine could lead to motor impairments.

Further research involving large-scale clinical data is necessary to elucidate the long-term effects of anesthesia drugs on children with CHD. This research will contribute to a better understanding of the risks associated with anesthesia exposure, inform clinical practice guidelines, and ultimately improve the care and outcomes of pediatric patients undergoing cardiac surgery.

## 12. Cardiopulmonary bypass factors

Prolonged CPB and deep hypothermia circulatory arrest can exacerbate cerebral embolism, inadequate cerebral perfusion, and hypoxic-ischemic injury, significantly impacting the neurodevelopment of children with CHD.^[52,53]^ Maiwald et al^[54]^ reported that the use of allopurinol during CPB can reduce the occurrence of ischemia-reperfusion injury, further decreasing the incidence of hypoxic brain injury and improving the long-term neurodevelopmental outcomes in children with CHD. However, further research is required to confirm the efficacy of allopurinol. Although studies comparing arterial blood gas management strategies (alpha-stat and pH-stat), hematocrit levels during CPB, blood glucose control levels, temperature management strategies, and comparisons between high-flow CPB, low-flow CPB, deep hypothermia circulatory arrest, and regional cerebral perfusion have not provided an optimal CPB management strategy,^[53]^ it is generally believed that using alpha-stat management during CPB may increase the risk of neurodevelopmental disorders in children with CHD postoperatively, and the hematocrit level during CPB should not be lower than 24%.^[55]^ Additionally, the use of prolonged ECMO and the increased incidence of complications such as sepsis, MODS, acute renal failure, and severe intracranial hemorrhage can lead to higher mortality rates in children with CHD and significant long-term neurodevelopmental delays in survivors.^[56]^

Optimizing CPB management strategies is essential for the treatment of children with CHD. By reducing the risk of complications, providing individualized treatment, continuous monitoring, and teamwork, surgical success rates and survival rates can be improved, thereby enhancing the long-term prognosis of these children.

## 13. ICU factors

DeWitt et al^[57]^ found in their study of 2419 neonates (≤28 days) and 10,687 non-neonates (39 days to 18 years) undergoing cardiac surgery and receiving ICU care that the threshold for prolonged ICU stay was 35 days for neonates and 10 days for non-neonates. Prolonged ICU stay affects the postoperative cognitive development of children with CHD,^[50]^ which may be related to the increased occurrence of systemic inflammation during ICU stay,^[58]^ prolonged endotracheal intubation duration, extended duration of vasopressor support, and delayed recovery of enteral nutrition support.^[59]^ Research aimed at identifying risk factors associated with prolonged ICU stays and developing targeted interventions to mitigate these risks can contribute to improved healthcare delivery and enhanced patient outcomes in the context of CHD management.

## 14. Other factors

In recent years, there have been a growing number of studies on the long-term neurodevelopment of children with CHD in relation to factors such as the educational level of parents and socioeconomic factors. Gaynor et al^[60]^ evaluated 1770 children with CHD (14.5 ± 3.7 months old) using the Bayley Scale for developmental assessment. The results suggested that children with higher maternal education levels had higher mental development index scores (88.2 ± 16.7), indicating better long-term neurodevelopment in children whose mothers had received undergraduate or higher education. Ozmen et al^[61]^ assessed 132 non-cyanotic children with CHD (35.2 ± 19.6 months old) using the Stanford–Binet Intelligence Scale and found that lower paternal education levels were associated with poorer intellectual development in children, while the correlation between maternal education levels and children’s intellectual development was less evident. Generally, the more complex the CHD surgery, the higher the cost required to sustain the child’s survival and development. von Rhein et al^[62]^ evaluated the intelligence of 117 school-aged children with CHD (10.4 ± 2.5 years old) and found that the IQ scores of children with CHD (89 ± 16) were significantly lower compared to normal children, with lower IQ scores only correlating with poorer socioeconomic conditions, highlighting the significant impact of socioeconomic factors on intellectual development in children with CHD.

Furthermore, studies on prenatal intrauterine brain development in fetuses with CHD,^[63]^ the choice of delivery mode and timing for CHD fetuses^[64]^ during pregnancy, the impact of perioperative thyroid hormones on neurodevelopment in children with CHD,^[65]^ and the use of serum biological markers to predict long-term neurodevelopment in children with CHD^[66,67]^ are increasing. von Werdt et al^[68]^ conducted a study involving 100 patients aged between 10 and 15 years of age who underwent complex CHD surgeries during the first year of life and found that the severity of stress in these children was significantly associated with impaired executive function. The interaction between stress stimuli and neurological systems impacting neuropsychological development requires further investigation.

By addressing not just the cardiovascular aspects but also the neurodevelopmental, psychological, and prenatal considerations, researchers aim to enhance the quality of life for children with CHD and improve their overall prognosis.

## 15. Neurodevelopmental Assessment Scales and monitoring methods

### 15.1. Assessment scales

The Bayley Infant Developmental Scale is an internationally recognized tool for the comprehensive assessment of infant development. It is suitable for infants aged 16 to 42 months and was first published in 1969, with subsequent updates in 1993, 2006, and 2019.^[69]^ The scale primarily includes assessments of cognitive, language, motor, social-emotional, and adaptive behavioral domains. Although the Bayley Scale is commonly used for neurodevelopmental assessment of children with CHD both domestically and internationally. Goldstone et al^[70]^ conducted Bayley-III assessments on 2198 children with CHD who underwent surgical treatment, and the results suggested that the Bayley-III scores were higher, potentially underestimating the prevalence of neurodevelopmental disabilities in children with CHD.

There are limited reports on neurodevelopmental assessments of children with CHD domestically. The Chinese Neurodevelopmental Scale for Children aged 0 to 6 years (Erxin Scale), based on the Bayley Scale, is widely used to evaluate children with autism domestically,^[71]^ its widespread use in children with CHD has not been extensively promoted.^[16]^

Currently, there is no unified standard for assessment scales, assessment systems, and follow-up systems that specifically target CHD populations, both domestically and internationally. In the future, it may be necessary to establish a risk assessment system tailored to children with CHD.

## 16. Monitoring methods

MRI can detect early anomalies in brain anatomy, focal or diffuse lesions, ischemic-hypoxic changes, and other conditions in children with CHD.^[72]^ 3D-MR imaging is more accurate in determining ventricular volume in children with CHD,^[73]^ whereas magnetic resonance spectroscopy can accurately reflect changes in brain microstructure and nutrient metabolism in children with CHD.^[74]^ Electroencephalogram can reflect the strength of electrical activity in the cerebral cortex and assess cognitive function with spatial resolution in specific areas.^[75]^ By recording electroencephalogram waveforms of children with CHD during sleep, cognitive function networks can be reflected, enabling the early identification of cognitive impairment.^[74]^ Near-infrared spectroscopy (NIRS) is commonly used to monitor intraoperative brain oxygen protection in children with CHD and promptly identify abnormal brain oxygen supply. Although the global utilization rate of NIRS is 74%,^[76]^ there are significant regional differences, and a consensus on ScO_2_ thresholds has not yet been reached.^[77]^ Furthermore, NIRS devices that can monitor the oxidation status of cytochrome c oxidase to reflect cell metabolism status are under development,^[78]^ which will bring revolutionary changes in monitoring brain oxygen and substance metabolism in children with CHD.

## 17. Conclusion

The factors leading to neurodevelopmental defects in children with CHD are complex and diverse. Precise surgical techniques, appropriate perioperative management strategy selection, and optimal monitoring methods can help improve neurodevelopment in children with CHD. Establishing a neurodevelopmental assessment and follow-up system for children with CHD is suitable for the early identification of risks affecting neurodevelopment, timely intervention, meeting long-term survival and developmental needs, and reducing the burden on families and society. This is of significant socioeconomic importance in countries with large populations such as China.

## Author contributions

**Supervision:** Shuantong Lin.

**Writing – original draft:** Shuantong Lin, Dequan Cao.

**Writing – review & editing:** Xiaojun Su, Shuantong Lin.
